# Translational applications of photopolymerizable hydrogels for cartilage repair

**DOI:** 10.1186/s40634-019-0215-3

**Published:** 2019-12-05

**Authors:** Weikun Meng, Liang Gao, Jagadeesh K. Venkatesan, Guanglin Wang, Henning Madry, Magali Cucchiarini

**Affiliations:** 1 Center of Experimental Orthopaedics, Saarland University and Saarland University Medical Center, Homburg/Saar, Germany; 2 Department of Orthopaedics, West China Hospital, West China School of Medicine, Sichuan University, Chengdu, Sichuan People’s Republic of China; 3 Department of Orthopaedic Surgery, Saarland University and Saarland University Medical Center, Homburg/Saar, Germany

**Keywords:** Photopolymerizable hydrogels, Articular cartilage, Tissue engineering, Mesenchymal stem cells, Chondrocytes, Biomolecules

## Abstract

**Background:**

Articular cartilage lesions generated by trauma or osteoarthritis are the most common causes of pain and disability in patients.

**Abstract:**

The development of photopolymerizable hydrogels has allowed for significant advances in cartilage repair procedures. Such three-dimensional (3D) networks of polymers that carry large amounts of water can be created to resemble the physical characteristics of the articular cartilage and be delivered into ill-defined cartilage defects as a liquid solution prior to polymerization in vivo for perfect fit with the surrounding native tissue. These hydrogels offer an adapted environment to encapsulate and propagate regenerative cells in 3D cultures for cartilage repair. Among them, mesenchymal stem cells and chondrocytes may represent the most adapted sources for implantation. They also represent platforms to deliver therapeutic, biologically active factors that promote 3D cell differentiation and maintenance for in vivo repair.

**Conclusion:**

This review presents the benefits of photopolymerization of hydrogels and describes the photoinitiators and materials in current use for enhanced cartilage repair.

## Background

Articular cartilage lesions remain a critical, unsolved problem in orthopaedics due to the inadequate capacity of this particular tissue for self-repair (Makris et al., 2015). While various options are available in the clinics, some of which promoting the restoration of hyaline cartilage in some patients, none of them satisfactorily lead to the generation of a repair tissue capable of withstanding mechanical stresses under natural conditions of weightbearing (Makris et al., 2015).

Strategies based on the use of a number of readily available biomaterials that are adapted for cartilage repair may provide valuable approaches to support and subsequently enhance the reparative activities in damaged cartilage (Cucchiarini & Madry, 2019). Such biomaterials must be both biocompatible and bioresorbable while supporting cell growth and differentiation, providing an adapted mechanical environment, and allowing for the transport of cell nutrients. Such systems include solid scaffolds, hydrogels, and hybrid materials of either natural or synthetic origin with specific advantages and limitations regarding their physical and mechanical properties (Cucchiarini & Madry, 2019).

Hydrogels as crosslinked hydrophilic polymers have attracted much attention with their ability to form 3D networks which can be fine-tuned to modify their biocompatibility and biodegradability (Hoffman 2012; Rey-Rico et al., 2016). Several types of hydrogels may be photopolymerized in the presence of photoinitiators using visible light (VL) or ultraviolet (UV) light and can be delivered as a liquid solution and then polymerized in vivo, allowing for a perfect fit between the hydrogel and the surrounding native tissue (Fedorovich et al., 2009). Controlled delivery of peptides, proteins, cells, and gene vectors may be achieved with the assistance of these hydrogels (Cucchiarini & Madry, 2019; Rey-Rico & Cucchiarini, 2016). In the present study, a systematic overview of the emerging photopolymerizable hydrogel-based treatments for cartilage repair is presented with the goal to address the unsolved problem of cartilage defects and to test the hypothesis that such therapeutic options may enhance the healing processes in sites of cartilage lesions.

## Current clinical approaches for cartilage repair

Patients with chondral and osteochondral lesions often experience joint pain, tissue swelling, and mechanical symptoms (e.g. locking, catching, or crepitus), which drive them to seeking treatment to relieve the secondary symptoms of joint disability (Grande et al., 2013). Cartilage lesions may be effectively managed with medical and conservative modalities. Yet, the incidence of cartilage degeneration and population ageing will result in more patients seeking for treatments for symptomatic joints. Current surgical treatment options are therefore expanding with new techniques being developed for specific age ranges and types of cartilage injuries (Hunziker, 2002).

### Non-surgical treatments

Non-surgical treatments are used to control the patients’ symptoms and disability and possibly slow the progression of the degenerative changes associated with the breakdown of the articular cartilage. Non-pharmacological treatments include patient education, physical therapy (e.g. heat and cold therapies), daily activity modification (e.g. weight reduction and non-weightbearing strengthening), bracing, orthotics, and non-irritating aerobic conditioning (Buttgereit et al., 2015). Pharmacological treatments include anti-inflammatory medication, possibly viscosupplementation and mild analgesics (e.g. acetaminophen), and intra-articular corticosteroid injections. In contrast to medication, exercise and weight loss have no side effects and may provide increases in range-of-motion and joint strength, chondroprotection, reduction of cartilage degradation and delayed progression to osteoarthritis (OA), without gastrointestinal, kidney, or liver toxicity. Yet, these options solely aim at alleviating pain.

### Surgical treatments

Surgical considerations for treating symptomatic defects include the etiology and chronicity, the general medical and systemic history of the patient, degree of containment, characteristics of defects, integrity of the meniscus and ligaments, and lower extremity alignment. When tissues, cells, and/or matrices are used to stimulate cartilage repair, regeneration, or replacement, they must be surgically adapted to the cartilage defect, with approximately 6 weeks of partial weightbearing to support for the biological switch to induce chondrogenesis in the defect. During this primary phase of biologic initiation and attachment in the new environment, protection of the forming repair tissue is desired to enhance the biologic incorporation. This may be accomplished with matrices, crosslinking, restricted motion, and limited surface forces (Simon & Jackson, 2018). Stabilization and retention of the early repair tissue within the cartilage defect are of great importance, however, if the implanted scaffold does not have structural or mechanical integrity to withstand the joint forces, adaptive and often degenerative changes may be observed in surrounding tissues of the defect (Jackson et al., 2001). Moreover, bleeding management is a critical consideration as blood and marrow cells may be selectively used in the repair. These host cells may compete with desired cells for populating a matrix. Attention must also be given to the convexity of the surfaces in two dimensions. As the depth and width of the compartment for the biologic material increases, the potential for a deleterious “zone of influence” on the area of surrounding native cartilage exists (Chen et al., 2011), including migration and thinning of adjacent cartilage and cyst formation within the subchondral bone (Peterson et al., 2003). In addition, iatrogenic injuries may occur from multiple sutures in the normal cartilage. Many procedures are technically demanding and may be applied only to specific patient populations; therefore, the objectives of the certain surgical technique must be clearly defined. Moreover, well-controlled prospective studies that demonstrate the role of the technique in cartilage repair, with either good or poor results, are needed.

#### Marrow stimulation

Marrow stimulation procedures establish a communication of the defect with the subchondral bone marrow compartment, allowing for migration of mesenchymal stem cells (MSCs) and subsequent chondrogenesis. The techniques include microfracture (Steadman et al., 2001), subchondral drilling (Gao et al., 2018), and abrasion arthroplasty (Johnson, 1986) which are relatively easy and cost-effective to perform. These techniques typically yield a filling of the defects with fibrocartilaginous repair rather than the original hyaline cartilage. Such a heterogeneous repair tissue may display inferior mechanical characteristics possibly leading to reduced long-term clinical outcomes.

#### Autologous chondrocyte implantation

The autologous chondrocyte implantation (ACI) technique extracts and cultivates articular chondrocytes ex vivo for transplantation to sites of cartilage lesions (Brittberg et al., 1994; Grande et al., 1987), allowing to advance the processes of cartilage repair in vivo. High success rates of cartilage repair were reported with this technique, yet ACI is not indicated for OA lesions, concomitant ligamentous instability, and abnormal weight distribution. Matrix-assisted ACI (MACI) has also been used as a means to grow autologous chondrocytes on a membrane scaffold which is implanted into the defect, replacing the covering of the defect with a periosteal flap (Tuan et al., 2013). Patients undergoing MACI in the knee show favorable mid- to long-term clinical outcomes, yet, a significantly higher treatment failure rate was found for defects in tibiofemoral joints versus those in patellofemoral joints (Schuette et al., 2017). Compared with microfracture, MACI has been shown to yield significantly improved 2-year outcomes for defects larger than 3 cm^2^ (Saris et al., 2014). In addition, autologous matrix-induced chondrogenesis (AMIC) is an alternative one-step procedure based on microfracture using defect coverage with a type-I/−III collagen matrix. Thus far, only one clinical study reported significant clinical improvements using AMIC for knee cartilage defects (mean defect size 3.6 cm^2^) versus microfracture after 5 years (Volz et al., 2017) but no studies systematically compared AMIC to MACI in the knee, to microfracture or ACI in the ankle, or to ACI in the hip.

#### Stem cell therapy

Stem cell-based therapy is an attractive approach to enhance cartilage repair, especially when applying MSCs that exhibit a reliable potential for chondrogenic differentiation. For instance, a multicenter randomized clinical trial comparing the arthroscopic transplantation of autologous bone marrow-derived MSCs via microfracture with microfracture alone in knee cartilage lesions revealed better osteochondral healing following cell therapy one year post-operatively (Hashimoto et al., 2019). A randomized controlled trial for the treatment of knee chondral lesions with peripheral blood MSCs-assisted arthroscopic microfracture/microdrilling reported significantly improved histological and radiographic cartilage restoration compared with microfracture/microdrilling (Saw et al., 2013). However, clinical administration of MSCs is hindered by a relative difficulty of harvesting, by alteration of cell phenotype over in vitro culture, and by a decreased differentiation capacity with age. Induced pluripotent stem cells (iPSCs) have been evoked as an alternative source of therapeutic cells as they can indefinitely proliferate while being available in large numbers and avoiding the limitations of MSC therapy. Still, the risk of teratoma formation following iPSC therapy should not be overlooked and strictly avoided prior to further initiation of such clinical trials. Large controlled studies are thus required to confirm whether such therapy can readily and safely improve clinical outcomes. Standardizing the manufacture and administration of cellular platforms and identifying adapted candidate treatments are necessary to determine their ultimate efficacy and to allow for strict comparisons with other established clinical approaches.

#### Osteochondral grafts

Such procedures are based on the transplantation of either osteochondral autografts or allografts. Both chondral and osteochondral lesions may be amenable to autograft transplantation, being rapidly covered with a mature, hyaline articular cartilage (Richter et al., 2016). Nevertheless, the procedure is technically challenging and associated with various complications, including recipient failure, subsidence of the graft surface and subchondral cyst formation as well as donor-site morbidity. Overall, autograft transplantation has been recommended to treat relatively small (chondral or osteochondral) defects (< 4 cm^2^). Recent evidence underlined the importance of selecting donor sites to optimize the contour of autograft and minimize the potential morbidity of harvest sites (Bartz et al., 2001). Owing to such limitations, allograft transplantation has recently gained increased attention (Dean et al., 2016), avoiding donor-site morbidity and allowing to treat large defects with mature articular cartilage in a single operation (Mirsasaani et al., 2011). However, the technique is restrained by the necessity of using fresh allografts (15–28 days) for optimal chondrocyte viability and mostly adapted for full thickness lesions.

#### Osteochondral scaffolds

Such compounds are employed to induce in situ regeneration via cells that originate from the bone marrow, leading to the formation of cartilage-like tissues. Several in vitro and in vivo studies reported noteworthy tissue formation even without additional cells (Kon et al., 2010) while a number of clinical trials demonstrated their benefits in terms of efficacy, safety, and mid-term satisfactory outcomes (Kon et al., 2014). A recent case series study reported that a cell-free collagen-hydroxyapatite osteochondral scaffold yielded short-term clinical improvements in treated patellar chondral defects (Perdisa et al., 2017). Clinical evidence also demonstrated that implantation of osteochondral scaffolds is well adapted for young patients, but also for individuals with early OA (Di Martino et al., 2015).

#### Metallic focal resurfacing implants

Treatments with metallic resurfacing implants provide a low friction bearing surface, achieve short-term symptomatic relief, but may complicate future interventions (Goebel et al., 2016) if used in younger patients.

## Advances in approaches using photopolymerizable hydrogels for cartilage repair

### Concepts of cartilage tissue engineering

As the current cartilage repair modalities are either too complex and invasive, or generate unsatisfactory outcomes, active investigation is ongoing to identify novel tools for more effective and convenient therapies of cartilage lesions. Tissue engineering offers the possibility to combine different therapeutic approaches based on cell and molecular biology, material science, and biomedical engineering to establish adaptable translational systems mimicking the normal cartilage structurally and mechanically. Starting with the input of the patient’s computed tomography (CT) data in a computerized tool system, it may be also envisaged to precisely print a combination of resources, resulting in a 3D construction mimicking the native articular cartilage tissue.

The most commonly used natural materials in cartilage research include agarose, alginate, chitosan, collagen, fibrin, and hyaluronan (Johnstone et al., 2013). Via specific surface receptors, these biomaterials may interact with cells to contribute to cell migration, production of extracellular molecules, and proliferation. Synthetic materials have been also extensively investigated including poly (ethylene glycol) (PEG), poly(N-isopropylacrylamide) (pNiPAAm), polylactide acid (PLA) and its derivatives (poly(L-lactic acid) - PLLA; poly (lactide-co-glycolide acid) - PLGA; poly(D,L-lactide acid) - PDLA), polyurethane (PU), and poly (vinyl alcohol) (PVA) (Johnstone et al., 2013). These polymers are relatively easy to produce, exhibiting suitable mechanical properties. Particularly, they exhibit a high potential to entrap living cells while providing a highly hydrated environment, facilitating nutrient diffusion, and serving as biological stimuli for cell migration, proliferation, and differentiation (Johnstone et al., 2013).

### Photopolymerizable hydrogels

Hydrogels, crosslinked hydrophilic polymers, represent an important class of biomaterials in biotechnology and biomedicine with excellent biocompatibility, causing minimal inflammatory responses, thrombosis, and tissue damage (Rey-Rico et al., 2016). Hydrogels can also swell large quantities of water without dissolution due to their hydrophilic but crosslinked structure. Additionally, hydrogels have high permeability for oxygen, nutrients, and other water-soluble metabolites. Over the past decades, a number of hydrogels differing in structure, composition, and properties have been developed and used extensively in medical applications such as contact lenses, biosensors, linings for artificial implants, and drug delivery devices (Ramakrishna et al., 2001).

**Fig. 1:**
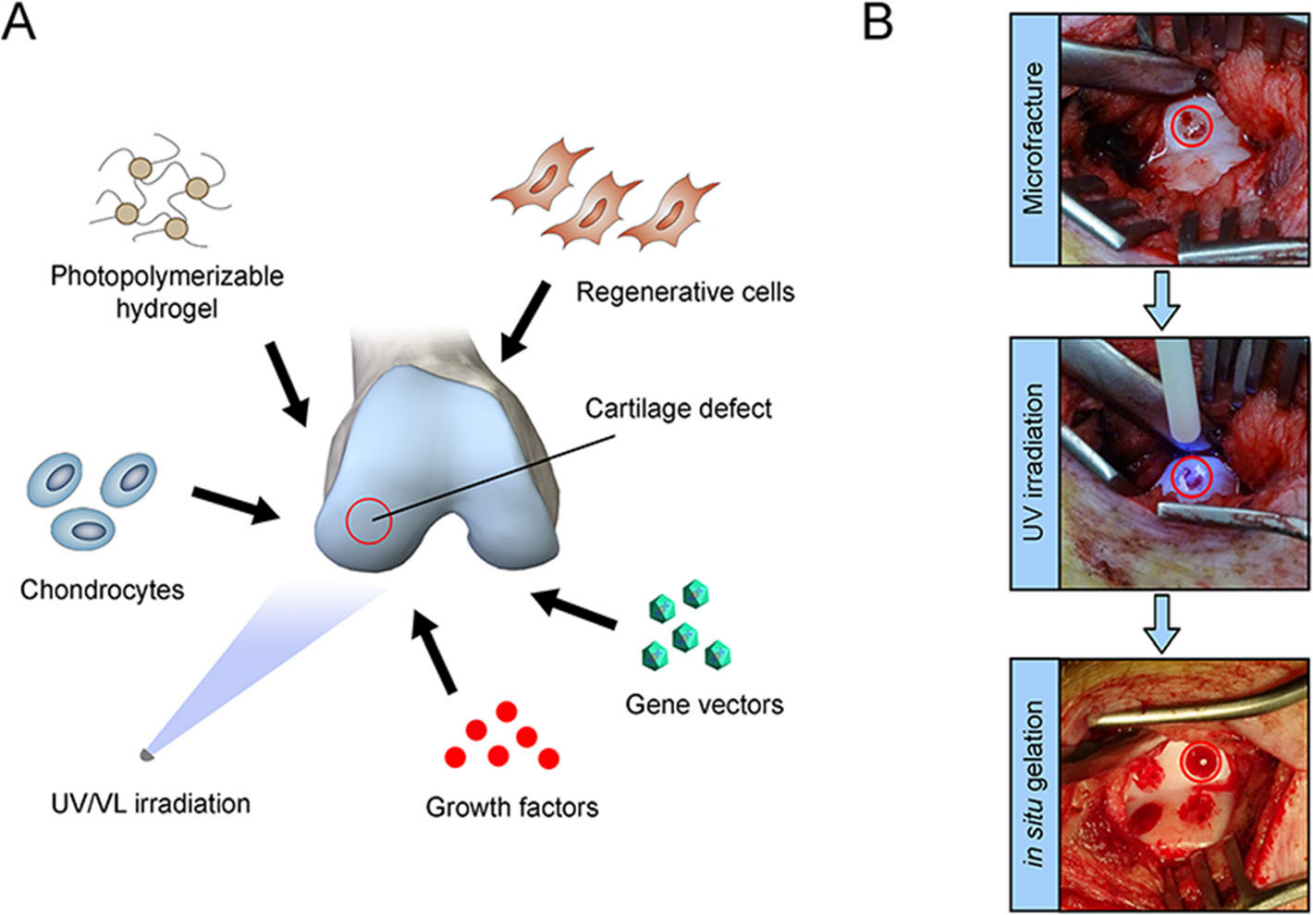
Application of photopolymerizable hydrogel systems for translational cartilage repair. (**a**) Principle of providing a photopolymerizable hydrogel carrying cells (regenerative cells, chondrocytes) and/or specific growth factors or gene vectors as controlled delivery platforms to sites of articular cartilage damage (hydrogel gelation can be performed ex vivo or in situ following radiation with ultraviolet - UV - or visible light - VL). (**b**) Intra-operative administration of a photopolymerizable hydrogel accompanied with microfracture into a focal full-thickness chondral defect allowing for in situ gelation via UV light polymerization

Some types of hydrogels can be photopolymerized in vivo and in vitro in the presence of photoinitiators using VL or UV light (Fig. 1) (Bryant et al., 2000). Photopolymerized hydrogels have been investigated for various biomedical applications including the prevention of thrombosis (Hill-West et al., 1994), postoperative adhesion formation (Hill-West et al., 1994; Hill-West et al., 1995), drug delivery (Peppas et al., 1999), cell transplantation (Elisseeff et al., 1999) and coatings for biosensors (An & Hubbell, 2000). VL or UV light can interact with light-sensitive compounds (photoinitiators) to create free radicals and trigger polymerization to form crosslinked hydrogels (Elisseeff et al., 1999). Photopolymerization has also been used in printing materials, membranes, polymeric materials, and surface coating/modifications (Johnstone et al., 2013). Photopolymerization has several advantages over conventional polymerization techniques, including a spatial and temporal control over polymerization, fast curing rates (less than a second to a few minutes) at room or physiological temperatures, and minimal heat production. In vivo photopolymerization has been extensively employed in dentistry to form sealant and dental restoration in situ (Mirsasaani et al., 2011), allowing also for in situ gelation from aqueous precursors in a minimally invasive manner via laparoscopic devices (Elisseeff et al., 2000), catheters (Ramakrishna et al., 2001), or subcutaneous injection with transdermal illumination (Elisseeff et al., 2000). Such applications, however, are difficult to perform with the narrow range of acceptable physiological temperature and pH environment as well as the general toxicity of most monomers and organic solvents. Some photopolymerization systems can overcome these limitations because the polymerization conditions are sufficiently mild (low light intensity, short irradiation time, physiological temperature, low organic solvent levels) to be carried out in the presence of cells and tissues.

### Applications in cartilage tissue engineering

#### Photopolymerizable hydrogel features

Photopolymerizable hydrogels are attractive for tissue engineering applications because of their high-water content and tissue-like mechanical properties. Their high water content mimics the permeability of the extracellular matrix (ECM) for optimal transport of oxygen, nutrients, and waste products, making them ideal for medical applications (Bryant et al., 1999). They can be delivered as a liquid solution and then polymerized in vivo for perfect fit with the surrounding native (cartilage) tissue (Fig. 1). Light-activated free radical crosslinking of hydrogels is of particular interest in cartilage repair strategies. Free radicals lead to the formation of covalent bonds between macromolecular precursor molecules in a rapid reaction that results in the formation of a polymer network with uniform and replicable physical properties. Long-wave UV light-activated polymerization is one of the most common methods of forming biomedical hydrogels with the advantages of temporal and spatial control of the reaction, low energy requirements, and clinically acceptable curing times (Baroli, 2006). Additionally, photopolymerizable hydrogels can be controlled to deliver peptides and proteins including growth factors by directly tethering the recombinant agents to the hydrogel for presentation to the embedded or surrounding cells. 3D hydrogels in cell therapy, for example, provide structural support for cells, enable proper diffusion of metabolites, and offer immune or local protection from host inflammation (Mironi-Harpaz et al., 2012).

Photopolymerizable hydrogels can be categorized according to their network structure, porosity, physical structure, source, and type of crosslinks (Johnstone et al., 2013). Crosslinks can be chemical or physical, with chemical crosslink being a covalent interaction at a point of overlap or junction and physical crosslink a physical entanglement of the polymer chains, interpenetrating polymer networks, and other secondary forces (Hill-West et al., 1995). According to their source, photopolymerizable hydrogels may be grouped as natural, synthetic, or hybrid (natural/synthetic) systems (Johnstone et al., 2013). Natural hydrogel constructs are often made of polysaccharide or protein chains. Polysaccharides have favorable hydrophilic structures allowing for the preparation of hydrogels (Rinaudo, 2008) like alginate, cellulose, chitin, chitosan, dextran, hyaluronic acid (HA), pectin, starch, and xanthan gum (Coviello et al., 2007; Yoshimura et al., 2006). Synthetic polymers such as PVA, polyacrylamide, poly (ethylene oxide) (PEO), and PEG have been used for hydrogel formation (Rey-Rico et al., 2016). Previous research confirmed that synthetic photopolymerizable hydrogels do not contain cell-binding motifs and are not biodegradable, thus support limiting cell proliferation and tissue integration. Synthetic photopolymerizable hydrogels are cell-compatible but often do not permit easy cell migration due to long lasting polymer components and are mechanically compromised (Lee et al., 2006; Roberts & Bryant, 2013). Natural polymers usually exhibit higher biocompatibility compared with synthetic polymers as they undergo enzyme controlled biodegradation by human enzyme-like lysozyme and produce biocompatible by-products (Sokker et al., 2009). Synthetic polymers are chemically stronger than natural ones because of hydrolyzable moieties with slower degradation rate. This feature provides more prolonged lifetime in the human body (Hoshikawa et al., 2006). Hybrid photopolymerizable hydrogels demonstrate higher cell viability and are injectable, biodegradable, and biocompatible, exhibiting excellent in situ space-filling qualities in air or aqueous solution without the use of protective barriers, while being resistant to swelling and contraction (Lin et al., 2019; Pascual-Garrido et al., 2019; Ramaswamy et al., 2008a; Sharma et al., 2007).

**Fig. 2:**
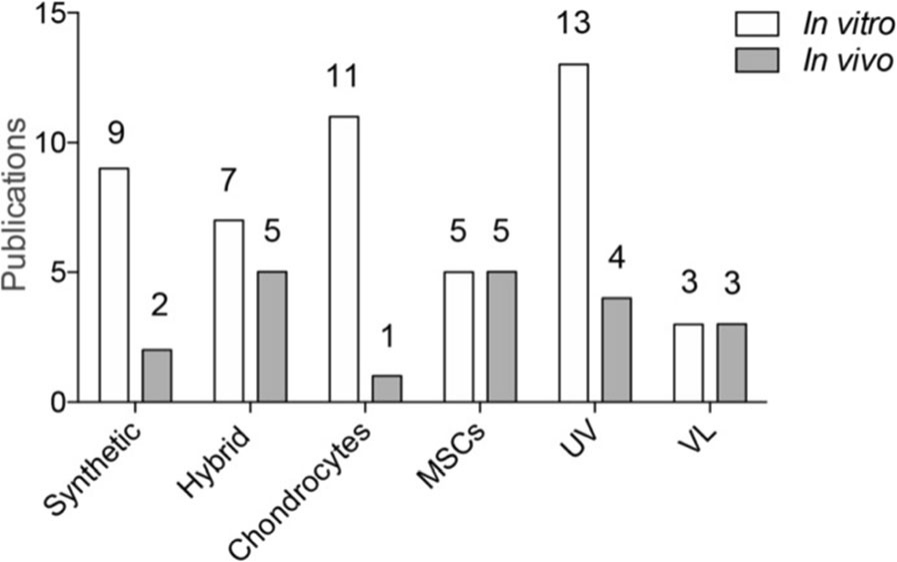
Current literature available on the use of photopolymerizable hydrogels in cartilage repair research in vitro and in vivo. MSCs, mesenchymal stem cells; UV, ultraviolet; VL, visible light

Thus far, 23 studies have reported the application of photopolymerizable hydrogels in cartilage research both in evaluations in vitro (Bryant & Anseth, 2001; Bryant & Anseth, 2002; Bryant & Anseth, 2003; Buxton et al., 2007; Elisseeff et al., 2000; Hayami et al., 2016; Hoshikawa et al., 2006; Kim et al., 2015; Lee et al., 2006; Levett et al., 2014; Lin et al., 2014; Neumann et al., 2016; Ramaswamy et al., 2008a; Roberts et al., 2011; Roberts & Bryant, 2013; Williams et al., 2003) (Fig. 2 and Table 1) and in vivo (Dua et al., 2016; Lin et al., 2017; Lin et al., 2019; Pascual-Garrido et al., 2019; Ramaswamy et al., 2008a; Schuette et al., 2017; Werkmeister et al., 2010) (Fig. 2 and Table 2). The methods of photopolymerization were based on the use of physical crosslinks, with UV light applied in 17 studies (Bryant & Anseth, 2001; Bryant & Anseth, 2002; Bryant & Anseth, 2003; Buxton et al., 2007; Dua et al., 2016; Elisseeff et al., 2000; Hayami et al., 2016; Lee et al., 2006; Levett et al., 2014; Lin et al., 2017; Neumann et al., 2016; Ramaswamy et al., 2008a; Ramaswamy et al., 2008b; Roberts et al., 2011; Roberts & Bryant, 2013; Sharma et al., 2007; Williams et al., 2003) and VL in 6 studies (Hoshikawa et al., 2006; Kim et al., 2015; Lin et al., 2014; Lin et al., 2019; Pascual-Garrido et al., 2019; Werkmeister et al., 2010). Both synthetic (Bryant & Anseth, 2001; Bryant & Anseth, 2002; Bryant & Anseth, 2003; Buxton et al., 2007; Elisseeff et al., 2000; Hoshikawa et al., 2006; Lin et al., 2014; Neumann et al., 2016; Ramaswamy et al., 2008a; Roberts & Bryant, 2013; Williams et al., 2003) and hybrid photopolymerizable hydrogels (Dua et al., 2016; Hayami et al., 2016; Kim et al., 2015; Lee et al., 2006; Levett et al., 2014; Lin et al., 2017; Lin et al., 2019; Pascual-Garrido et al., 2019; Ramaswamy et al., 2008b; Roberts et al., 2011; Sharma et al., 2007; Werkmeister et al., 2010) have been employed and their use and application are described below.

**Table 1:** Use of photopolymerizable hydrogels in vitro for applications in cartilage research

Hydrogels	Factors	Photoinitiators				Cells	Follow-up	Results	References
		Type	Wavelength (nm)	Time (min)	Intensity (mw/cm^2^)				
PEODM^a^	–	UV	365	10	10	calf chondrocytes	6 weeks	Maintained cell viability, uniform cell seeding, ECM is not compromised as the scaffold thickness is increased from 2 to 8 mm	(Bryant & Anseth, 2001)
PEGDM^a^ PEG-LA-DA^b^	–	UV	365	20	10	calf chondrocytes	6 weeks	Chondrocytes encapsulated in hydrogels with at least 75% degradable crosslinks produced a cartilaginous tissue, increased type-II collagen synthesis	(Bryant & Anseth, 2003)
				10			weeks	Production of similar biochemical matrix content to newly synthesized cartilaginous tissue, high compressive modulus to restore function and increase type-II collagen synthesis	(Buttgereit et al., 2015)
PEG/PEGDA^a^	–	UV	–	4-10	–	hBMSCs	6 weeks	Greater PEGDA molecular weight did not affect proteoglycan content at a PEGDA:PEG ratio of 2:1 but did affect the distribution, addition of PEG to PEGDA hydrogels resulted in greater collagen content, facilitated cell chondrogenesis	(Buxton et al., 2007)
PEO/PEODM^a^	–	UV	–	3	2-3	bovine, ovine chondrocytes	2 weeks	Chondrocyte survival and dispersed cell population composed of ovoid and elongated cells, proteoglycan and collagen contents increases over 2 weeks of static incubation, functional ECM with equilibrium moduli, dynamic stiffness, and streaming potentials that increased with time	(Elisseeff et al., 2000)
MGC/MCS^b^ MHA/MCS^b^		UV	320–390	1	10	bovine chondrocytes	5 weeks	Improved cell viability and matrix production (aggrecan, type-II collagen), only MHA/MCS hydrogels retain an approximately constant modulus	(Hayami et al., 2016)
Styrenated gelatin^a^	–	VL	400–520	2	800	rabbit chondrocytes	2 weeks	Maintained chondrogenic phenotype and cell viability	(Hoshikawa et al., 2006)
Gel-MA^b^ Gel-HA^b^ Gel-CS^b^ Gel-HA-CS^b^	–	UV	365	15	2.6	human chondrocytes	8 weeks	Enhanced chondrogenesis and mechanical properties	(Levett et al., 2014)
PEG-CAP^a^ PEG-CAP-NOR^a^	–	UV	352	7	–	bovine chondrocytes	4 weeks	Increased sGAGs and collagen in the hydrogels with time, type-II collagen and aggrecan present in the neotissue with formation of a territorial matrix beginning at day 21, 8-fold increase in compressive modulus from days 7 to 28	(Neumann et al., 2016)
PEODA^a^	–	UV	365	7	3-4	bovine chondrocytes	7 weeks	MRI-derived measurements of matrix FCD in injectable hydrogels reflect tissue GAG content	(Ramaswamy et al., 2008a)
PEG-LA^b^	–	UV	365	10	6	bovine chondrocytes	4 weeks	Degradation led to 2.3- and 2.9-fold greater GAG and collagen contents compared with PEG group, respectively, macroscopic cartilage-like tissue formation (aggrecan, type-II and -VI collagen, link protein, and decorin) but decreased moduli, retention of the chondrocyte phenotype, proteoglycan and type-II collagen deposition	(Roberts et al., 2011)
PEGDA^a^ PEGTNB/PEGDSH^a^	–	UV	352	10	6	bovine chondrocytes	2 weeks	PEGTNB led to hyaline-like cartilage production especially under mechanical loading, polymerization mechanism and network structure have long-term effects on the quality of engineered cartilage, especially under mechanical loading	(Roberts & Bryant, 2013)
MeGC/Col^b^	TGF-β1	VL	400–500	120	500–600	hSMSCs	3 weeks	TGF-β1 controlled release, maintained cell viability and chondrogenesis, cell aggregation and ECM deposition most particularly in the presence of TGF-β1 and type-II collagen impregnation relative to pure MeGC hydrogels	(Kim et al., 2015)
mGL/LAP^a^	TGF-β3	VL	430-490	2, 4, 8	1400	hBMSCs	13 weeks	High viability and chondrogenic differentiation of encapsulated cells	(Lin et al., 2014)
PEG/PEGDA^a^	TGF-β1	UV	365	5	4	goat BMSCs	6 weeks	Effective cell chondrogenesis, enhanced by TGF-β1	(Williams et al., 2003)
PEODA/CMP^b^	CMP	UV	365	5	5	bovine chondrocytes	2 weeks	Maintained cell viability, production of type-II collagen, CMP provides cell-manipulated crosslinks and collagen binding sites that simulate natural ECM	(Lee et al., 2006)

^a^Synthetic photopolymerizable hydrogels; ^b^natural/synthetic (hybrid) photopolymerizable hydrogels. *PEO* poly (ethylene oxide), *PEODM* PEO-dimethacrylate, *PEG* poly (ethylene glycol), *PEGDM* PEG-dimethacrylate, *PEG-LA-DA* poly (lactic acid)-b-poly (ethylene glycol)-b-poly (lactic acid) endcapped with acrylate groups, *PEGDA* PEG-diacrylate, *MGC* N-methacrylate glycol chitosan, *MCS* O-methacrylate chondroitin sulfate, *MHA* O-methacrylate hyaluronic acid, *Gel-MA* gelatin-methacrylamide, *Gel-HA* gelatin-hyaluronic acid, *Gel-CS* gelatin-chondroitin sulfate, *Gel-HA-CS* gelatin-hyaluronic acid-chondroitin sulfate, *PEG-CAP* PEG-caprolactone, *PEG-CAP-NOR* PEG-CAP endcapped with norbornene, *PEODA* PEO-diacrylate, *PEG-LA* poly (lactic acid)-b-poly (ethylene glycol)-b-poly (lactic acid), *PEGTNB* PEG-tetranorbornene, *PEGDSH* PEG-dithiol, *MeGC* methacrylated chitosan, *Col* collagen, *mGL* methacrylated gelatin, *LAP* lithium phenyl-2,4,6-trimethylbenzoylphosphinate, *CMP* collagen mimetic peptide, *TGF-β* transforming growth factor beta, *UV* ultraviolet, *VL* visible light, *MSCs* mesenchymal stem cells, *hBMSCs* human bone marrow-derived MSCs, *hSMSCs* human synovium-derived MSCs, *ECM* extracellular matrix, *sGAGs* sulfated glycosaminoglycans, *MRI* magnetic resonance imaging, *FCD* fixed charge density

**Table 2:** Use of photopolymerizable hydrogels in vivo for applications in cartilage research

Hydrogels	Factors	Photoinitiators				Cells	Model	Follow-up	Results	References
		Type	Wavelength (nm)	Time (min)	Intensity (mw/cm^2^)					
PEODA/HA^a,b^	–	UV	–	7	4–5	hBMSCs	rabbit (full-thickness chondral defects)	4 weeks	Cartilage repair after 28 days, enhanced cellularity of de novo tissues that filled the defects	(Dua et al., 2016)
MeHA^b^	–	UV	360	–	1.2	rat BMSCs	mice (full-thickness chondral defects)	8 weeks	MeHA is biocompatible and osteoconductive, no sign of chondrocyte aggregation in the defects	(Lin et al., 2017)
mGL/MHA^b^	–	VL	430–490	4	1400	hBMSCs	rabbit (full-thickness osteochondral defects)	12 weeks	MSC chondrogenesis, optimal cartilage and bone formation using mGL/MHA at 9:1	(Lin et al., 2019)
PEG/MMP-2^b^	–	VL	352	8	5	rabbit BMSCs	rabbit (full-thickness osteochondral defects)	24 weeks	MSC chondrogenesis in vivo	(Pascual-Garrido et al., 2019)
PEODA/HA^b^	–	UV	365	5	6–8	–	rabbit (full-thickness chondral defects)	5 weeks	Cartilage repair	(Ramaswamy et al., 2008b)
PEG/PCL^a^	–	VL	450	1	1000	human chondrocytes	rat (s.c.)	4 weeks	ECM deposition (type-II and -VI collagen, GAGs), cartilage repair	(Werkmeister et al., 2010)
PEODA/HA^b^	TGF-β3	UV	365	10	4	hBMSCs	mouse (s.c.)	3 weeks	Chondrogenic differentiation	(Sharma et al., 2007)

^a^Synthetic photopolymerizable hydrogels; ^b^natural/synthetic (hybrid) photopolymerizable hydrogels. *PEODA* poly (ethylene oxide) diacrylate, *HA* hyaluronic acid, *MeHA or MHA* methacrylated HA, *mGL* methacrylated gelatin, *PEG* poly (ethylene glycol), *MMP-2* matrix metalloproteinase 2, *PCL* poly(ε-caprolactone), *TGF-β* transforming growth factor beta, *UV* ultraviolet, *VL* visible light, *MSCs* mesenchymal stem cells, *hBMSCs* human bone marrow-derived MSCs, *s.c.* subcutaneous, *ECM* extracellular matrix, *GAGs* glycosaminoglycans

#### Photopolymerizable hydrogels as cell supportive matrices for cartilage repair

Photopolymerizable hydrogels have been manipulated to target MSCs (Buxton et al., 2007; Dua et al., 2016; Kim et al., 2015; Lin et al., 2014; Lin et al., 2017; Lin et al., 2019; Pascual-Garrido et al., 2019; Sharma et al., 2007; Williams et al., 2003) and chondrocytes (Bryant & Anseth, 2001; Bryant & Anseth, 2002; Bryant & Anseth, 2003; Elisseeff et al., 2000; Hayami et al., 2016; Hoshikawa et al., 2006; Lee et al., 2006; Levett et al., 2014; Neumann et al., 2016; Ramaswamy et al., 2008a; Roberts et al., 2011; Roberts & Bryant, 2013; Werkmeister et al., 2010) for applications in cartilage research in vitro (Bryant & Anseth, 2001; Bryant & Anseth, 2002; Bryant & Anseth, 2003; Buxton et al., 2007; Elisseeff et al., 2000; Hayami et al., 2016; Hoshikawa et al., 2006; Kim et al., 2015; Lee et al., 2006; Levett et al., 2014; Lin et al., 2014; Neumann et al., 2016; Ramaswamy et al., 2008a; Roberts et al., 2011; Roberts & Bryant, 2013; Williams et al., 2003) (Fig. 2 and Table 1) and in vivo (Dua et al., 2016; Lin et al., 2017; Lin et al., 2019; Pascual-Garrido et al., 2019; Ramaswamy et al., 2008b; Sharma et al., 2007; Werkmeister et al., 2010) (Fig. 2 and Table 2).

In vitro, Bryant & Anseth (Bryant & Anseth, 2001; Bryant & Anseth, 2002; Bryant & Anseth, 2003) reported that encapsulated chondrocytes produced a cartilaginous tissue using poly (lactic acid)-b-poly (ethylene glycol)-b-poly (lactic acid) endcapped with acrylate groups (PEG-LA-DA), with increased type-II collagen synthesis. Buxton et al. (Buxton et al., 2007) demonstrated that higher poly (ethylene glycol diacrylate) (PEGDA) molecular weight affected the distribution of proteoglycans and that addition of PEG in PEGDA hydrogels resulted in greater collagen contents. Elisseeff et al. *(Elisseeff et al.,* 2000*)* showed that chondrocytes encapsulated in a PEO diacrylate (PEODA) hydrogel exhibited increased proteoglycan and collagen contents and equilibrium moduli, dynamic stiffness, and streaming potentials. Hayami et al. (Hayami et al., 2016) reported that N-methacrylate glycol chitosan (MGC)/O-methacrylate chondroitin sulfate (MCS) and O-methacrylate HA (MHA)/MCS hydrogels improved the production of matrix compounds in chondrocytes. Hoshikawa et al. *(Hoshikawa et al.,* 2006*)* noted that chondrocytes in styrenated gelatin using camphorquinone as a photoinitiator displayed steady expression of type-II collagen and aggrecan core protein mRNAs. Levett et al. (Levett et al., 2014) showed that chondrocytes in gelatin-methacrylamide (Gel-MA) hydrogels had improved mechanical properties with addition of HA-MA or CS-MA. Neumann et al. *(Neumann et al.,* 2016*)* observed that the use of PEG-based photopolymerizable hydrogels increased sulfated glycosaminoglycans (sGAGs) and collagens in the newly formed tissue. Ramaswamy et al. (Ramaswamy et al., 2008a) demonstrated that chondrocytes in PEODA hydrogels increased their GAG contents at high (> 75%) level of viability. Roberts et al. (Roberts et al., 2011; Roberts & Bryant, 2013) showed that PEG-tetranorbornene (PEGTNB) led to long-term hyaline-like cartilage production under mechanical loading.

In vivo, Dua et al. (Dua et al., 2016) demonstrated that the presence of hydroxyapatite particles enhanced the cellularity in the repair tissue in defects treated with microfracure and cell-free PEGDA, accelerating remodeling. Lin et al. (Lin et al., 2017) described synergistic effects of chondrogenic preconditioning and mechanical stimulation on bone marrow-derived MSCs in methacrylated HA (MeHA) hydrogels, with superior chondrogenic differentiation in rat osteochondral defects. Lin et al. (Lin et al., 2019) reported that methacrylated gelatin (mGL)/MHA enhanced the regeneration of the osteochondral unit in rabbit full-thickness osteochondral defects. Pascual-Garrido et al. *(Pascual-Garrido et al.,* 2019*)* noted that MSCs undergo effective chondrogenesis in a cartilage-mimetic hydrogel using PEG with matrix metalloproteinase 2 (PEG/MMP-2) that can be delivered in vivo and photopolymerized intra-operatively in situ. Ramaswamy et al. (Ramaswamy et al., 2008b) reported that sealing full-thickness chondral defects with a PEODA hydrogel yielded a repair tissue comparable to the surrounding normal cartilage in rabbits. Werkmeister et al. (Werkmeister et al., 2010) employed a PEG/poly(ε-caprolactone) (PEG/PCL) system to generate the formation of an ECM rich in type-II collagen and GAGs in rats.

#### Photopolymerizable hydrogels as controlled delivery systems of agents for cartilage repair

Growth and development of cartilage tissue relies heavily on biochemical signals. The sequence, duration, and intensity of stimulation can all play roles in how cells secrete matrix in a regenerating environment. Bioactive molecules can include growth factors, adhesion proteins, peptide sequences, or any other agent that binds to cells to create a biological response. Numerous studies showed that these growth factors can also elicit dramatic changes in articular chondrocytes.

The transforming growth factor beta (TGF-β) is a classical factor used in cartilage engineering studies, leading to the stimulation of chondrogenesis and proliferation (Blunk et al., 2002) although in some cases to an inhibition of matrix formation (Verschure et al., 1994). In vitro, Kim et al. (Kim et al., 2015) described that controlled delivery of TGF-β1 using methacrylated chitosan (MeGC) hydrogels enhanced cellular aggregation and deposition of cartilaginous ECM by the encapsulated cells (Table 1). Lin et al. *(Lin et al.,* 2014*)* showed that a photopolymerized mGL hydrogel was capable of supporting MSC growth and TGF-β3-induced chondrogenesis. Williams et al. (Williams et al., 2003) demonstrated that a PEG-based hydrogel allows for the chondrogenic differentiation of MSCs in the presence of TGF-β1*.* In vivo, Sharma et al. (Sharma et al., 2007) demonstrated that MSCs in hydrogels containing both HA and TGF-β3 produced high quality cartilage (Table 2). HA enhanced proteoglycan production when combined with TGF-β3 and reduced the production of type-I collagen.

The collagen mimetic peptide (CMP) is a less extensively studied molecule that is expressed almost exclusively in cartilage (Choi et al., 1983), binding to aggrecan and type-II collagen while the chondrocytes attach to it via α1β1 integrin. When used as a coating material, CMP enhanced both cell attachment and spreading on surfaces (Makihira et al., 1999). The addition of type-II collagen to the CMP coating showed even more improvement in these characteristics. In vitro, Lee et al. (Lee et al., 2006) reported that CMP-PEODA hydrogels stimulated the production of GAGs and collagen in chondrocytes and suggested that high levels of ECM in such systems were due to the affinity of CMP to chondrocyte-secreted collagen, allowing for a collagen-rich environment ideal for further ECM production.

## Challenges and outlooks of the use of photopolymerizable hydrogels for translational cartilage repair

Due to their biocompatibility, permeability, and physical characteristics, photopolymerizable hydrogels are promising candidates for use in minimally invasive cartilage repair procedures with a one-step in situ functionalization. Equally important, they can be locally applied via arthroscopy, making them highly valuable for ease of manipulation and to avoid potential problems associated with open surgery such as high physical impact, increased risks for complications, and extended recovery time. They may also be combined with current clinical options like marrow stimulation and ACI, a concept supported by the current application of non-photopolymerizable hydrogel compounds via such procedures. Such hydrogels can be functionalized as improved spatio-temporal controlled delivery systems of bioactive (chondroreparative) agents, potentially protecting such “cargo” from physiological degradation. Photopolymerizable hydrogel-guided delivery of gene therapy vectors may thus be also envisaged as a potential tool to promote the effective, long-lasting healing of damaged articular cartilage (Cucchiarini & Madry, 2019). This very innovative concept may also have the advantage to mask viral capsid epitopes when using virus-derived vectors that may otherwise trigger undesirable toxic and/or immune responses in joint tissues (Cucchiarini & Madry, 2019).

However, a number of specific issues and challenges remain that need to be carefully addressed for the optimal use of photopolymerizable hydrogels in the goal of translational cartilage research. First, while the use VL has minimal deleterious effects, UV light exposure may be damaging to cellular DNA in the surrounding tissues, potentially leading to accelerated tissue aging and oncogenic activation. For improved biosafety, application of VL at wavelengths of 450–550 nm could be an alternative source of light for the crosslinking of photopolymerizable hydrogels. Such VL may deeply penetrate tissues with relatively low energy, making it optimal for the development of in situ injectable hydrogels for in vivo applications in a minimally invasive manner. Next, evidence showed that synthetic photopolymerizable hydrogels do not contain cell binding motifs and are not biodegradable, supporting limited cell proliferation and tissue integration. These compounds also do not permit cell migration due to long lasting polymer components and are mechanically compromised. Such issues may be addressed by using natural/synthetic (hybrid) photopolymerizable hydrogels, nevertheless, very limited work has been performed thus far with hybrid systems for applications in cartilage repair.

## Conclusions

Cutting-edge evidence has advanced our general knowledge on the feasibility of using photopolymerizable hydrogels for cartilage research. Yet, more work is needed to demonstrate the potential benefits of these systems as convenient, adapted, and safe systems for the treatment of cartilage defects. First, current experimental work both in vitro and in clinically relevant animal models in vivo has to be expanded in order to define optimal conditions for effective therapy (hydrogel class and nature; presence, source, type, and dose of reparative cells, bioactive agent(s), and/or gene vector). Also, the initiation of clinical trials where various, optimized photopolymerizable hydrogels will be applied in patients is of the utmost importance to provide effective systems suitable for improved cartilage repair in translational approaches in a close future.
